# Oropharyngeal dysphagia in patients with multiple sclerosis: do the disease classification scales reflect dysphagia severity?

**DOI:** 10.5935/1808-8694.20130082

**Published:** 2015-10-08

**Authors:** Alessandro Murano Ferré Fernandes, André de Campos Duprat, Cláudia Alessandra Eckley, Leonardo da Silva, Roberta Busch Ferreira, Charles Peter Tilbery

**Affiliations:** a Instructor/Professor - Department of Otorhinolaryngology, Santa Casa de São Paulo. MSc and PhD in Medicine from the School of Medical Sciences of Santa Casa de São Paulo. Instructor in the Department of Otorhinolaryngology, Santa Casa de São Paulo.; b Instructor/Professor - Department of Otorhinolaryngology, Santa Casa de São Paulo MSc and PhD from the School of Medical Sciences of Santa Casa de São Paulo. Instructor, Department of Otorhinolaryngology, Santa Casa de São Paulo.; c Assistant Professor - Department of Otorhinolaryngology, Santa Casa de São Paulo. MSc and PhD from the School of Medical Sciences of Santa Casa de São Paulo. Assistant Professor, Department of Otorhinolaryngology, Santa Casa de São Paulo.; d MSc in Neurosciences from the Federal University of São Paulo - Paulista School of Medicine - Clinical Speech and Hearing Therapist (Clinical Speech and Hearing Therapist).; e Professor in the Department of Neurology at the Santa Casa de São Paulo (Professor, Department of Neurology at the Santa Casa de São Paulo). Department of Otorhinolaryngology, Santa Casa de São Paulo.

**Keywords:** impairment assessment, multiple sclerosis, swallowing disorders

## Abstract

Multiple sclerosis is a neurological disease that involves swallowing disorders. Many studies have shown an association between neurological and swallowing performance, but results have been conflicting.

**Objective:**

To identify the frequency of dysphagia in patients with multiple sclerosis and neurological indicators that can represent the performance of swallowing.

**Method:**

In this study (cross-sectional) 120 Multiple Sclerosis patients underwent Functional Assessment of Swallowing by flexible nasal-pharyngo-laryngoscopy and the results were compared with the scores of the rating scales: (Clinical Evolving Forms of Disease, Functional Disability Scale for and Scale Systems Extended Functional Disability [Kurtzke Expanded Disability Status Scale]).

**Results:**

Dysphagia was found in 90% of patients. Among the clinical forms of the disease, the progressive forms (primary progressive and secondary progressive) were more frequently associated with severe dysphagia, while the relapsing-remitting form presented more often mild and moderate dysphagia. Regarding the Disability Scale for Functional Systems, cerebellar function, brainstem function and mental health were associated with dysphagia, especially in the severe form. Regarding the Extended Functional Disability Scale, higher scores were associated with severe dysphagia.

**Conclusion:**

Dysphagia is common in MS patients, especially in those with greater impairment of neurological functions.

## INTRODUCTION

Multiple sclerosis (MS) is a disease of probable autoimmune origin, characterized by inflammation of the central nervous system, demyelination and gliosis formation. The combination of genetic, immunological and infectious factors seems to explain its appearance. It most often affects young individuals, especially Caucasian women between 20 and 40 years of age, being rare in Asians and blacks, and unusual in individuals older than these age limits^1^.

The distribution of this disease in the globe is not uniform. Countries like England, New Zealand, the northern United States, southern Canada and Australia are highly prevalent, with more than 30 patients per 100,000 inhabitants. Brazil and the other countries of South America have low prevalence rates; however, the mixing of races with European influence caused an increased prevalence in the states of São Paulo and Minas Gerais, with rates of around 18 patients per 100,000 inhabitants^1^.

Clinically, MS is characterized by a variable combination of symptoms such as limb weakness, gait disorders, sensitivity disorders, ataxia, and visual changes. Demyelination can be abrupt or have an insidious onset and variable severity. Neurological signs may present from very faint to obvious changes and limit patient activities. The deficits may undergo total remission or have sequels and, over time, all patients tend to show progressive neurological restrictions. Treatment for disease control is done primarily with corticosteroids (used especially during worsening) and immunomodulators^1^, ^2^.

Swallowing-associated disorders, although not much investigated, may be part of the clinical manifestations. Studies investigating these changes show variable frequencies of appearance, with scores lying between 10 and 100%, contributing to disease morbidity and mortality^3^, ^4^, ^5^, ^6^, ^7^, ^8^, ^9^, ^10^, ^11^.

The concern in identifying patients with this functional change and prevent its complications has stimulated researchers to find neurological indicators that represent the performance of swallowing. The association between dysphagia and MS rating scales (Clinical Evolving Forms of Disease, Functional Disability Scale Extended - EDSS [Kurtzke's Expanded Disability Status Scale] and Functional Disability Scale Systems - EIFS) has been reported by some studies; however, there is no consensus and there are controversies regarding the methods and the results obtained^4^, ^5^, ^6^, ^8^, ^12^, ^13^, ^14^, ^15^, ^16^, ^17^, ^18^, ^19^.

The difficulty in assessing the swallowing of all patients with MS makes us seek those with higher risk of dysphagia, especially the more susceptible to aspiration and the development of lower airways infection. Thus, the aim of this study was to evaluate the prevalence of dysphagia in patients with MS and identify neurological indicators that can represent swallowing performance.

## METHOD

From January 2007 to May 2011 we evaluated 120 patients (95 women and 25 men) with MS in a tertiary university hospital. This study was approved by the Ethics in Human Research under protocol #039/06.

The age ranged between 17 and 65 years with a mean of 38.5 (median = 38, SD = 10.2). Patients were diagnosed and classified according to the Evolutionary Clinical Forms of the disease, with the Functional Disability Scale by Systems (EIFS) and with the EDSS - Kurtzke's Expanded Disability Status Scale. The study excluded patients who were having acute demyelination; those with other neurological disorders not associated with MS, and those anatomical changes in the oral cavity, pharynx and larynx that might interfere with swallowing. We also excluded those patients with tracheostomy or a history of having been submitted to it.

The Evolving Clinical Forms of MS are divided into relapsing-remitting (RR), in which the patient has flares and remissions of neurological deficit, in general, with good recovery; primary progressive (PP), in which the disease progresses from the beginning with slowly progressive neurologic deficit; and a secondary progressive (SP) - initially similar to the relapsing-remitting form, but after a while, it becomes progressive. Of the total 120 patients, 79 (65.8%) had RR; 35 (29.2%) had SP; and six (5%) had PP. The SP and PP forms, due to their similar clinical behavior, were grouped and called progressive forms (PF).

The EIFS classifies the deficit according to the neurological system evaluated. It analyzes the pyramidal, sensory, cerebellar, bladder, brain stem, intestinal, visual, mental and other dysfunctional systems^1^. Pyramidal dysfunction was observed in 108 patients (90%); sensory dysfunction in 76 (63.3%); cerebellar dysfunction in 63 (52.5%); bladder dysfunction in 54 (45%); brainstem dysfunction in 42 (35%); bowel dysfunction in 41 (24.2%); visual dysfunction in 34 (28.3%); mental dysfunction in 21 (17.5%) and other disorders in 18 (15%).

EDSS classifies patients according to their neurological disability considering gait and EIFS^1^. The score starts at 0, which represents no disability and reaches 9.5, which represents complete disability. The score is set at 10 for patients who died. The evaluation revealed that the EDSS score ranged from 0 to 9, with an average of 3.9 points (median = 3.5, SD = 2.5). In this study we did not have any patient with a score of 9.5.

After neurological classification, the patients were submitted to Swallowing Video-Endoscopic (VED) assessment, following the protocol described by Langmore et al.^20^. During its execution, the patients were seated in the examination ENT chair as usual, in a wheelchair or in bed with the head raised in 45 degrees. The flexible nasal-laryngoscope was inserted through the wider nasal cavity of the patient, without the use of vasoconstrictors or local anesthetics, to avoid interference in sensitivity and swallowing.

The equipment used for the test was the flexible Pentax 3.6 mm fiber optic scope, with a Micro-camera-CAM DX Storz NTSC model 202301 20, Machida Model RG-2500 Light source, LG 21 inch TV, DVD recorder LG DR model 4912B.

During this exam we gave the patients soft foods (10 ml in a tablespoon), fluids (5 ml and 10 ml in a syringe) and solids (piece of biscuit of salt and water type) stained with blue food dye (aniline dye) and at room temperature so as to prevent thermal stimulation of the regions tested during the examination.

We used a clinical and functional scale for the laryngoscopy findings to determine the severity of dysphagia^21^. In this classification, the changes found must occur in at least one of the tested food types:

• Normal swallowing - normal oral retention of the foodstuff (no food escape before and after the pharyngeal phase of swallowing), no food stasis, laryngeal penetration or tracheal aspiration;
• Mild dysphagia - mild stasis after swallowing food (stasis taking up less than a third of the area of the epiglottic vallecula and piriform recesses), absence of laryngeal penetration or tracheal aspiration;
• Moderate dysphagia - moderate food stasis after swallowing (stasis of foodstuff taking up about two-thirds of the epiglottic vallecula and piriform recesses), laryngeal penetration of food, but no tracheal aspiration;
• Severe dysphagia - important food stasis after swallowing (stasis of food that fully occupies the region of the epiglottic vallecula and piriform recesses) and tracheal aspiration.

After characterizing the severity of dysphagia by VED findings, the data was correlated with the clinical forms of the disease (RR and PF) and the scales of functional disability (EDSS and EIFS).

This study was carried out through descriptive statistics using means, medians, standard deviations and percentages. A comparison of various qualitative variables was performed using the chi-square test or Fisher's exact test, depending on the expected values in contingency tables. We compared the EDSS score mean values using the Mann-Whitney and Kruskal-Wallis test, using the Tukey HSD test for comparisons between groups (post hoc test). For all statistical tests we used a significance level of 5%.

## RESULTS

Swallowing performance assessment showed that 108 patients (90%) had abnormalities and only 12 (10%) were normal in this task. Among those patients with swallowing disorders, 49 (40.8%) had mild dysphagia, 44 (36.7%) moderate dysphagia and 15 (12.5%) severe dysphagia.

**Figure 1:**
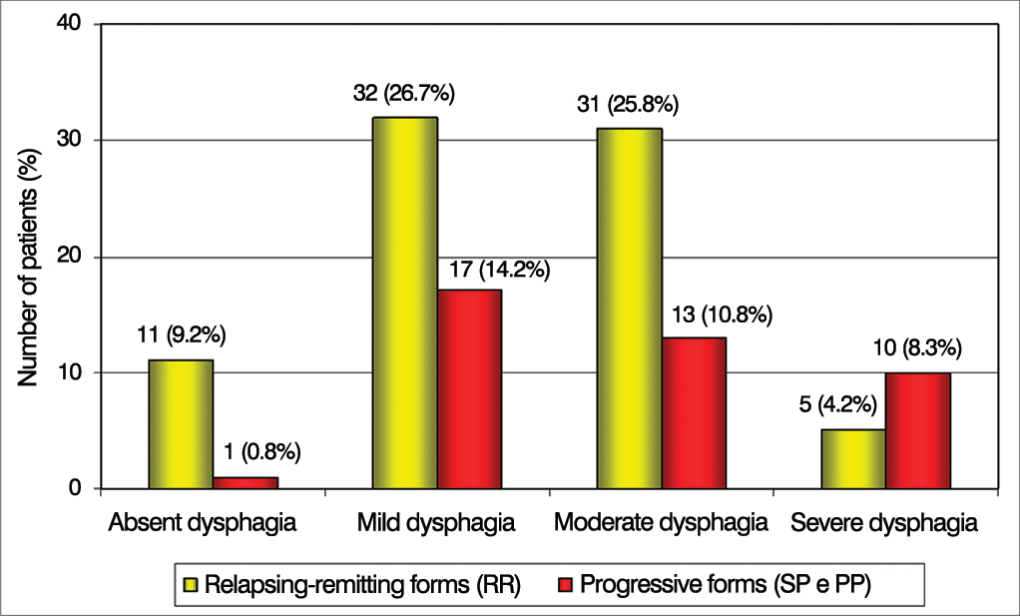
Patient distribution according to the severity of dysphagia and the Clinical Presentations of Multiple Sclerosis.

To establish the influence of the clinical evolution of MS on the swallowing behavior we made a comparison between this variable and dysphagia severity. Of patients with severe dysphagia, the progressive forms (SP and PP) were more frequent, while mild and moderate dysphagia occurred more frequently in RR (*p* = 0.033) patients (Figure 1).

**Figure 2:**
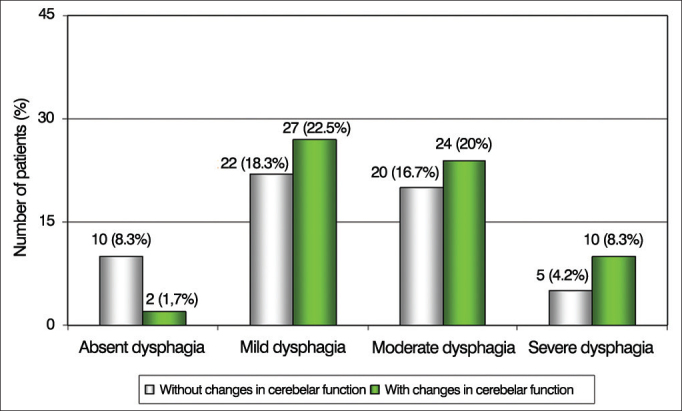
Patient distribution according to the severity of dysphagia and changes in cerebellar function in the Functional Disability Scale by Systems (EIFS).

Regarding the presence of dysphagia and neurological disability defined by EIFS, there was a statistically significant association with altered cerebellar function (*p* ≤ 0.05). Stratified analysis showed statistically significant differences between this brain function vis-à-vis patients with the mild, moderate and severe dysphagia. The results are shown in Figure 2.

**Figure 3:**
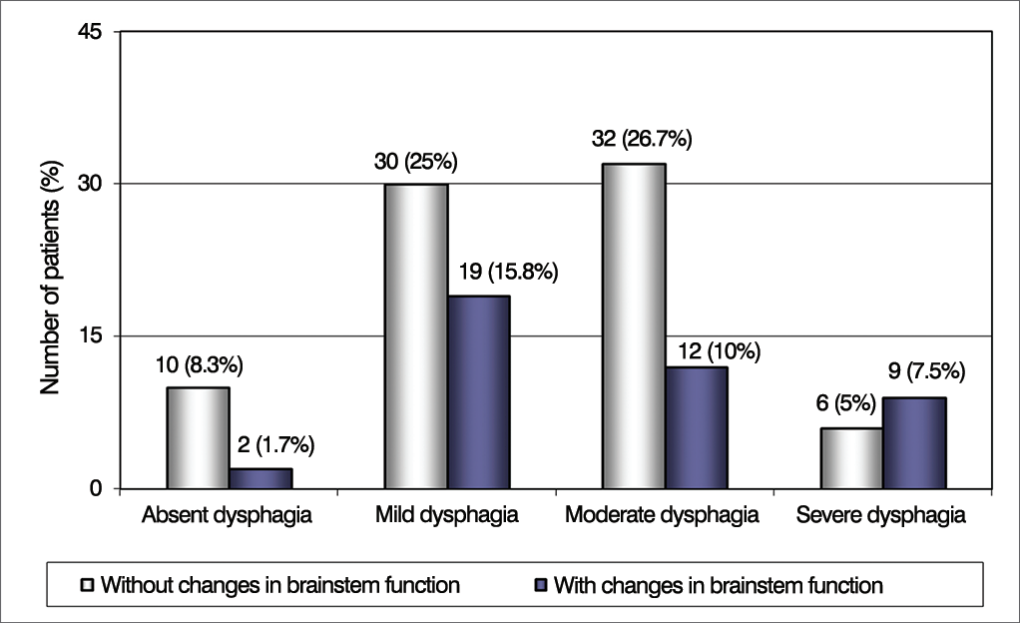
Patient Distribution according to the severity of dysphagia and changes in brainstem function in the Functional Disability Scale by Systems (EIFS).

**Figure 4:**
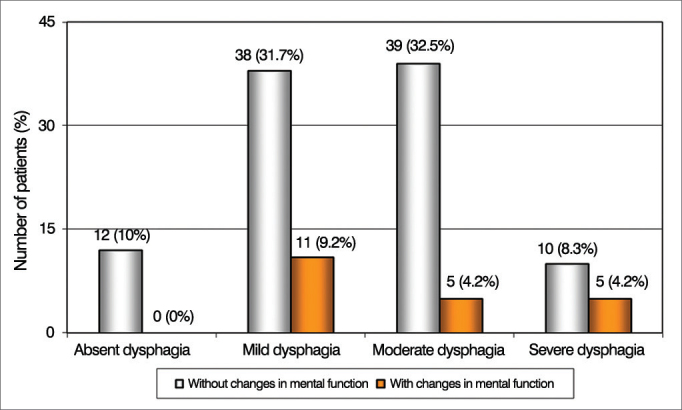
Patient distribution according to the severity of dysphagia and changes in mental function in the Functional Disability Scale by Systems (EIFS).

We also noticed that only patients with severe dysphagia were influenced by changes in brainstem and mental functions, and were significantly more numerous (Figures 3 and 4). Other neurological functions showed no statistically significant association with the degree of dysphagia.

**Figure 5:**
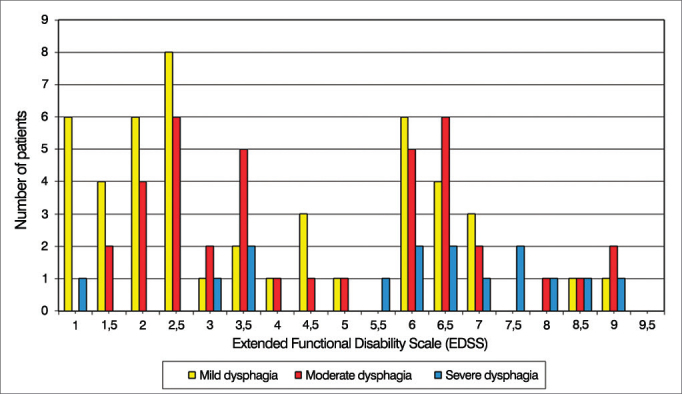
Patient distribution according to the severity of dysphagia and scores on the Extended Functional Disability Scale (EDSS).

Swallowing performance was also compared with the EDSS score. We observed that patients with mild dysphagia had lower EDSS scores, averaging 3.6 points (SD = 2.4). Patients with moderate dysphagia had slightly higher scores, averaging 4.1 points (SD = 2.6) and patients with severe dysphagia exhibited the highest scores, averaging 5.9 points (SD = 2.3). The Kruskal-Wallis test revealed a statistically significant difference in EDSS mean values, according to the degree of dysphagia (*p* = 0.007). The post hoc test showed differences between the groups of patients with moderate versus severe dysphagia (*p* = 0.037) and severe versus mild dysphagia (*p* = 0.005) (Figure 5).

## DISCUSSION

Multiple Sclerosis is a disease that causes progressive neurologic deficits, with changes in the sensory and motor systems, with a potential functional impact on swallowing. In this study we used the VED as an evaluation method, which allowed us to examine patients confined to a hospital bed, with a deficit in movement and postural changes^22^, ^23^, ^24^. This form of investigation has full agreement with the videodeglutogram (or videofluoroscopy) and is easier to execute, without exposing patients to radiation^21^, ^25^, ^26^, ^27^, ^28^, ^29^, ^30^, ^31^.

Most patients in this study (90%) had dysphagia disorders that occurred during the evolution of the disease. These high rates agree with the studies from Miani et al., Abraham & Yun, Wiesner et al. and Terre-Boliart et al. and contrast with studies that report dysphagia in amounts ranging between 10 and 50%^3^, ^5^, ^11^, ^16^, ^18^.

The need to identify patients potentially prone to dysphagia has led some researchers to study the factors representing the performance of this task. The study on the influence of the disease's progression impact on swallowing performance showed that patients with the progressive forms (SP and PP) were in greater number of individuals with severe dysphagia compared to RR patients. The progressive forms, because of their more destructive and permanent behavior, cause sequelae that led to more severe restrictions on swallowing performance.

Besides comparisons with clinical forms of MS, the association of dysphagia with scales of disability (EDSS and EIFS) has also been used to infer performance. The EIFS evaluates neural systems and brain structures affected by MS. The functions performed by the pyramidal system, cerebellum, brainstem, sensory system and areas related to mental activity change the dynamics of swallowing and can cause drastic changes in the performance of pharyngeal and laryngeal structures. The pyramidal function was the most frequently affected among the patients analyzed in this study and others; however there was no significant association with the occurrence of dysphagia. In contrast, cerebellar, mental and brainstem functions showed significant levels of influence, a fact also noted by authors who conducted similar studies^4^, ^5^, ^8^, ^10^, ^12^, ^13^, ^14^, ^15^, ^16^, ^18^. During motor tasks, the cerebellum operates in sequencing muscle activity, coordinating the agonist and antagonist muscles so there is no competition. Moreover, it acts in the anticipation of movements to be performed by storing the programming needed for a given task. This storage extends throughout life, and whenever a similar task is performed, this programing is reused^32^, ^33^. In swallowing, the sequence of events related to muscle activity and contraction speed and force are important for proper food propulsion, and changes to cerebellar activity can cause great damage.

Brainstem and mental functions also cause influences, though its occurrence was determined only in patients with severe dysphagia. The brainstem, owner of the nuclei that control swallowing, and the brain structures linked to mental function, predominantly located in the frontal lobe, responsible for initiative, judgment, attention, and cognition, clearly interfere with the performance of this activity and can cause significant misfits^32^, ^34^.

The EDSS analysis also allows inferences on swallowing performance, and higher scores on this scale are associated with a greater impairment in this task^6^, ^10^, ^15^, ^17^. Likewise, in this study the mean EDSS score increased progressively as dysphagia worsened, being higher in patients with dysphagia classified as severe. These findings are in disagreement with the ones from Abraham & Yun^4^, who found no relationship between EDSS and dysphagia severity, which may have happened due to the small sample size of that study (13 patients)^4^. However, Abraham et al.^15^ (1997) questioned 538 patients and found that the severity of dysphagia was related to the EDSS score. In this study, the most intense manifestations of dysphagia were more frequent in patients with major neurological damage and higher EDSS scores.

Therefore, the observation of Evolutionary Clinics Forms of the disease, EIFS evaluation - especially of mental neurological, cerebellar and brainstem functions, and EDSS are useful to infer about possible changes in swallowing and stimulate research by objective methods vis-à-vis this task.

## CONCLUSION

Swallowing disorders assessed by swallowing video endoscopy were frequent in this group of patients with Multiple Sclerosis. The clinically progressive forms of the disease (SP and PP forms), the involvement of cerebellar, brainstem and mental functions on the Functional Disability Scale by Systems (EIFS) and high scores on the Extended Functional Disability Scale (EDSS) were factors associated with dysphagia severity in such patients.
